# Few geographic and socioeconomic variations exist in primary total shoulder arthroplasty: a multi-level study of Australian registry data

**DOI:** 10.1186/s12891-016-1134-4

**Published:** 2016-07-16

**Authors:** Sharon L. Brennan-Olsen, Richard S. Page, Stephen E. Lane, Michelle Lorimer, Rachelle Buchbinder, Richard H. Osborne, Julie A. Pasco, Anita E. Wluka, Kerrie M. Sanders, Peter R. Ebeling, Stephen E. Graves

**Affiliations:** School of Medicine, Deakin University, Geelong, Australia; Australian Institute for Musculoskeletal Sciences, The University of Melbourne, St Albans, Australia; Institute for Health and Ageing, Australian Catholic University, Melbourne, Australia; Barwon Orthopaedic Research Unit, Barwon Health, Geelong, Australia; Barwon Health Biostatistics Unit, Barwon Health, University Hospital, Geelong, Australia; School of BioSciences, University of Melbourne, Melbourne, Australia; Australian Orthopaedic Association Joint Replacement Registry, Adelaide, Australia; Department of Epidemiology and Preventive Medicine, Monash University, Alfred Centre, Melbourne, Australia; Monash Department of Clinical Epidemiology, Cabrini Hospital, Malvern, Australia; School of Health and Social Development, Deakin University, Melbourne, Australia; NorthWest Academic Centre, Department of Medicine, The University of Melbourne, St Albans, Australia; Department of Medicine, Monash University, Melbourne, Australia; Epi-Centre for Healthy Aging, IMPACT Strategic Research Centre, Deakin University, (Barwon Health), PO Box 281, Geelong, VIC 3220 Australia

**Keywords:** Arthroplasty, Socioeconomic factors, Shoulder joint, Geographic locality, Australia

## Abstract

**Background:**

Associations between socioeconomic position (SEP) and the uptake of primary total shoulder arthroplasty (TSA) is not well understood in the Australian population, thus potentially limiting equitable allocation of healthcare resources. We used the Australian Orthopaedic Association National Joint Replacement Registry (AOA NJRR) to examine whether geographic or socioeconomic variations exist in TSA performed for a diagnosis of osteoarthritis 2007–11 for all Australians aged ≥40 years.

**Methods:**

Primary anatomical and reverse TSA data were extracted from the AOA NJRR which captures >99 % of all TSA nationally. Residential addresses were cross-referenced to Australian Bureau of Statistics 2011 Census data to identify SEP measured at the area-level (categorised into deciles), and geographic location defined as Australian State/Territory of residence. We used a Poisson distribution for the number of TSA over the study period, and modelled the effects of age, SEP and geographic location using multilevel modelling.

**Results:**

During 2007–11, we observed 6,123 TSA (62.2 % female). For both sexes, TSA showed a proportional increase with advancing age. TSA did not vary by SEP or geographic location, with the exception of greater TSA among men in New South Wales.

**Conclusions:**

Using a national registry approach we provide the first reliable picture of TSA at a national level. The uptake of TSA was equitable across SEP; however, there was some variation between the States/Territories. With an aging population, it is imperative that monitoring of major surgical procedures continues, and be focused toward determining whether TSA uptake correlates with need across different social and area-based groups.

## Background

Shoulder osteoarthritis (OA), while not considered to be life threatening, is a degenerative disease that is associated with increased pain, reduced function, and loss of motion [1]. For patients with end-stage glenohumeral OA, total shoulder arthroplasty (TSA) of the humeral head and glenoid is a well-established treatment method [2]. Positive clinical results and reduction in pain have been reported post-TSA in observational studies [3–8]. Interestingly, and in absence of data from controlled trials of surgery *vs.* placebo or nonsurgical interventions [9], to date, best clinical practice for shoulder OA remains unknown. Nevertheless, similar to the weight-bearing joints (hip or knee), individuals affected by shoulder OA may suffer far-reaching functional consequences if surgery is delayed [10]. Whilst some data suggests the number of TSAs due to arthritis will increase during the coming years due to severe disease in our ageing populations [2, 11–13], advances in nonsurgical treatment, especially for rheumatoid arthritis, may act in the opposite direction and potentially decrease or delay the need for TSA.

Similar to other chronic diseases, a social gradient of arthritis has been observed in higher income countries [14, 15]. In Australia, we recently reported a 42 % increased likelihood of self-reported arthritis (at any skeletal joint) for those of greater social disadvantage compared to those who are more advantaged [14]. Those differences were independent of advancing age and female sex: risk factors that are key predictors of OA [1, 16]. Associations have been observed between SEP and surgical intervention for severe end-stage OA of the hip or knee in England [17–21], USA [22], Italy [23] and Australia [24], and geographic differences in hip or knee replacements have also been observed [25–29]. In comparison to arthroplasty of large skeletal joints (hip or knee), there is a paucity of data regarding whether differences exist in TSA across SEP or geographical regions, especially in Australia. The associations between TSA and different social and area-based groups are important to understand as it may indicate inequality of healthcare provision or uptake. To address the paucity of Australian data in this field of enquiry, and in order to inform the evidence-base regarding the uptake of shoulder joint replacement across socioeconomic and geographic strata, we aimed to present the first data pertaining to TSA utilization rates across SEP and geographic location in adults, using data from the comprehensive Australian national joint replacement registry 2007–11.

## Methods

### Australian Orthopaedic Association National Joint Replacement Registry

The Australian Orthopaedic Association National Joint Replacement Registry (AOA NJRR), one of only six nationally based shoulder registries available worldwide [30], commenced documenting knee and hip joint replacement surgeries in September 1999. Data collection was introduced in a State-by-State approach that was completed nationally in 2002 [31]. In 2004, the AOA NJRR was expanded to include shoulder replacement surgeries, with full national level data collection commencing in November 2007. Similar expansion occurred during the same decade for joint replacement registries held in New Zealand and Scandinavian countries [30]. The AOA NJRR monitors the performance and outcome of joint replacements Australia-wide and receives voluntary cooperation from all hospitals undertaking joint replacement surgeries performed within both the public and private health systems. Data are matched and verified by cross-linking registry data with government hospital separation data for all arthroplasty procedures. This verification process has established that the Registry receives information on more than 99 % of all joint replacement surgeries. The AOA NJRR is the most complete and extensive set of joint replacement data in Australia, and has been validated against health department unit record data using a sequential multi-level matching process coupled with the retrieval of unreported procedures [31].

For this study, incident primary TSA performed 2007–11 was defined as primary replacement of the diseased or damaged ‘ball’ (proximal head of the humerus) and ‘socket’ (glenoid) of the shoulder joint due to OA. We also included reverse TSA; a similar procedure to the primary replacement but whereby the prosthetic ‘ball and socket’ are anatomically reversed, to improve the biomechanics particularly when the rotator cuff tendons around the shoulder are deficient. Revision surgeries, hemi-arthroplasty and TSA for other conditions such as rheumatoid arthritis were not included.

### Socioeconomic position

The full residential address of each patient undergoing a TSA performed during 2007–11 was matched to the corresponding 2011 Australian Bureau of Statistics (ABS) Statistical Area Level 1 (SA1), an area of approximately 250 households, and subsequently classified into the corresponding Local Government Area (LGA), as described below. ABS reference data were used to determine the Socio-Economic Indexes For Areas (SEIFA) value from the 2011 census for each TSA at the LGA level. We applied the Index of Relative Socioeconomic Disadvantage (IRSD) for this analysis, in which decile 1 represented the most disadvantaged and decile 10 represented the least disadvantaged. Validation of the SEIFA index was undertaken by analysts from the ABS Regional Offices and also an external peer review of the variables and methodology used in SEIFA 2006 was performed by a group of academic and policy research experts who were skilled in socioeconomic modelling and analysis [32]. Variables included in the SEIFA were validated by summing SEIFA variables at the small area to the State totals, and principal components analysis used to develop and weight the scores [32, 33]. In 2011, approximately 4 % of census collection districts could not be given a SEIFA score by the ABS due to low population density in areas, or poor data quality [34]. Further, some addresses could not be geocoded with sufficient precision to match against census boundaries, leading to SEIFA values being unavailable for 7 % of the patients in the AOA NJRR dataset that had undergone TSR, and given that imputation would not be possible, these patients were excluded from this analysis.

### Local Government Areas (LGAs) and State/Territory of residence

From the cross-referencing process of residential addresses to the ABS census data for 2011, we also ascertained the LGA and the State or Territory in which each patient resided. Using ABS concordance files, patient data was further mapped to LGAs, so that full age by sex population counts could be used. LGAs are administrative boundaries that cover incorporated areas that are legally designated parts of States/Territories over which incorporated local governing bodies have responsibility [35]. Across Australia there are 568 LGAs, encompassed within the 6 States (New South Wales, Queensland, South Australia, Western Australia, Tasmania and Victoria), and 2 Territories (Australian Capital Territory and Northern Territory).

### Age groupings

Patients were categorised into 10-year age groupings for analyses. TSA was performed on a small number of patients in the 20–29 and 30–39 year age groups (*n* = 2 and *n* = 7, respectively). Those patients were excluded from analyses as it was deemed inappropriate to include data cells that contained small numbers as this would have increased the likelihood of model non-convergence.

### Statistical analysis

Multilevel modelling was used to estimate the effect of area-level SEP, LGA and State/Territory of residence on the uptake of TSA. Given that the prevalence of shoulder OA is greater among females, we planned to analyse males and females separately a priori. Further, due to the structure of the population data from the ABS, age and sex groupings were required to be aggregated at the LGA level. As such, the number of TSA in each sex and age group within the LGA boundaries were aggregated over the study period.

We used a Poisson distribution for the number of TSA over the study period, as inspection of the data at various levels of aggregation showed considerable skew. Poisson regression also allowed us to model the counts directly. Effects of age, LGA, area-level SEP and State/Territory of residence were modelled using a multilevel model, assuming vague priors on all coefficients; an offset was allowed in the model for the population at risk in each LGA over the study period. Results were presented as rate per 10,000 person years (py), which was the expected number of TSA in 10,000 persons over a one year period. Specific model details are as follows:

Denoting *y*_*i*_ as the number of TSA for the *i*^th^ age group by LGA aggregation, and following the notation of Gelman and Hill [36], the model considered was:

$$ \begin{array}{l}{y}_i\sim \mathrm{Poisson}\left({\lambda}_i\right)\\ {}{\lambda}_i= \exp \left( \log {N}_i+\mu +{a}_{j\left[i\right]}^{\mathrm{age}}+{\alpha}_{k\left[i\right]}^{\lg \mathrm{a}}+{\eta}_i\right)\\ {}{\alpha}_j^{\mathrm{age}}\sim N\left(0,{\sigma}_{\mathrm{age}}^2\right)\\ {}{\alpha}_k^{\lg \mathrm{a}}\sim N\left({\alpha}_{l\left[k\right]}^{\mathrm{ses}}+{\alpha}_{m\left[k\right]}^{\mathrm{state}},{\sigma}_{\lg \mathrm{a}}^2\right)\\ {}{\alpha}_l^{\mathrm{ses}}\sim N\left(0,{\sigma}_{\mathrm{ses}}^2\right)\\ {}{\alpha}_m^{\mathrm{state}}\sim N\left(0,{\sigma}_{\mathrm{state}}^2\right)\\ {}{\eta}_i\sim N\left(0,{\sigma}_{\eta}^2\right)\end{array} $$

Where *α*_*j*_^age^ and *α*_*k*_^lg a^ are parameters to be estimated for the age and LGA indicators respectively. LGA is further modelled at the group-level by including indicators for SEP and geographic location (State/Territory). All variance parameters were modelled with uniform (0, 10) priors.

A Bayesian approach was employed to estimate the parameters in the model, with all models generated using Stan [37], and summaries calculated in R Version 3.0.2 [38]. Models were assessed using graphical posterior predictive checks, and convergence was assessed by the Gelman-Rubin diagnostic [39]; results are reported as (log) coefficients and 95 % posterior intervals (95 % PI). Finally, we calculated estimates and 95 % credible intervals (95 % CI) in terms of the difference in log (coefficients) for each SEP decile relative to SEP decile 1 (most disadvantaged). The AOA NJRR Data Review Committee approved this study.

## Results

During the years 2007–11, 6,123 TSA surgeries (62.2 % female) were performed within 562 of the 568 LGAs. Unadjusted rates of TSA showed a positive association with advancing age; surgeries peaked at 3 per 10,000 py for men in the 70–79 year age group and 4.7 per 10,000 py for females in the same age group (data not shown).

**Fig. 1:**
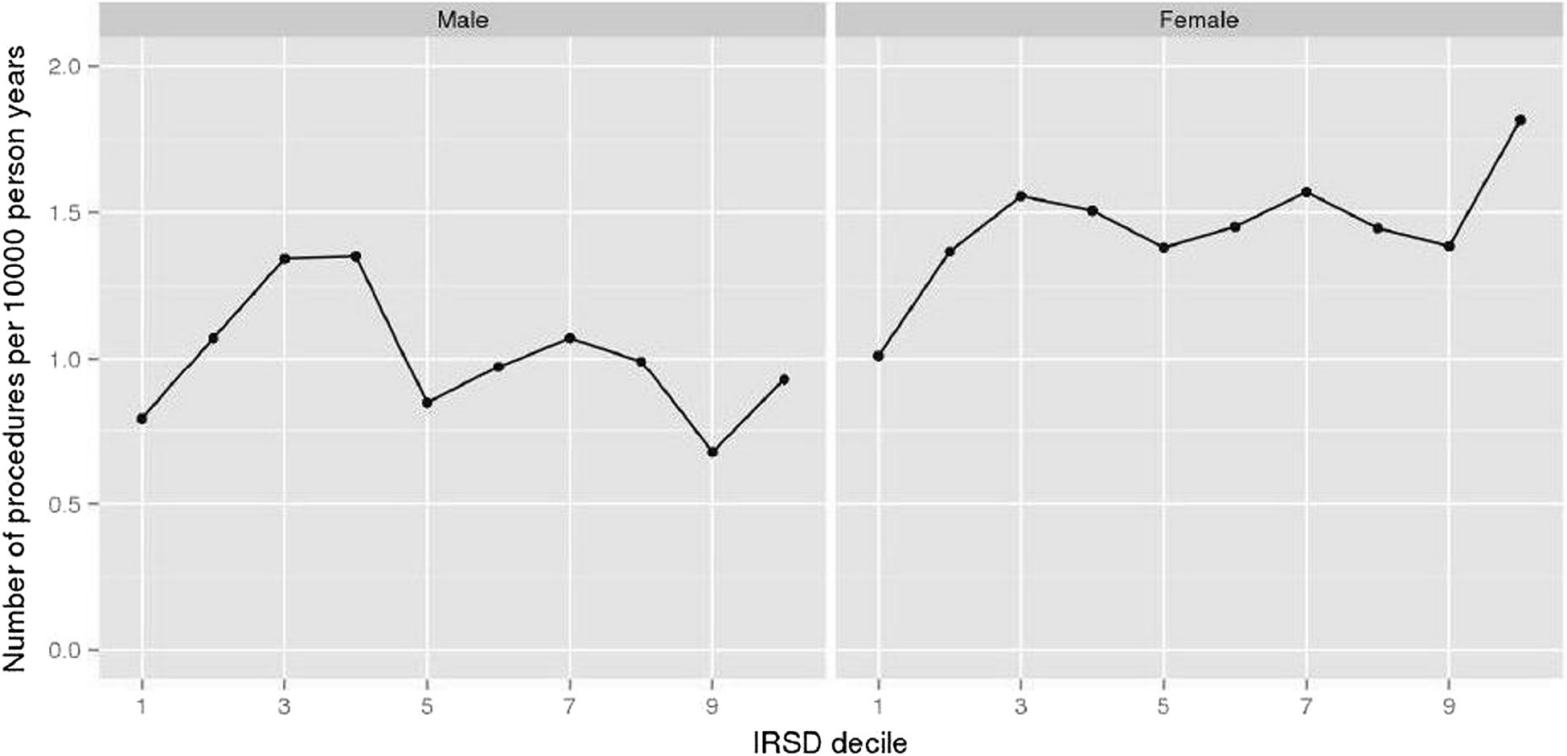
Unadjusted rates of primary total shoulder arthroplasty per 10,000 person years in males and females across deciles of socioeconomic position (SEP) measured by the Index of Relative Socioeconomic Disadvantage (IRSD). SEP decile 1 is the most disadvantaged

Figure 1 presents unadjusted rates of TSA per 10,000 py by SEP decile and sex. For men, unadjusted TSA differed across SEP deciles, although this was not linear; ranging from ~0.75 per 10,000 py in SEP decile 1 (most disadvantaged) to ~0.90 per 10,000 py in SEP decile 10 (least disadvantaged). The unadjusted rate of TSA for females also increased non-linearly as social disadvantage decreased; TSA ranged from ~1.00 per 10,000 py in SEP decile 1 to ~1.85 per 10,000 py in SEP decile 10.

**Fig. 2:**
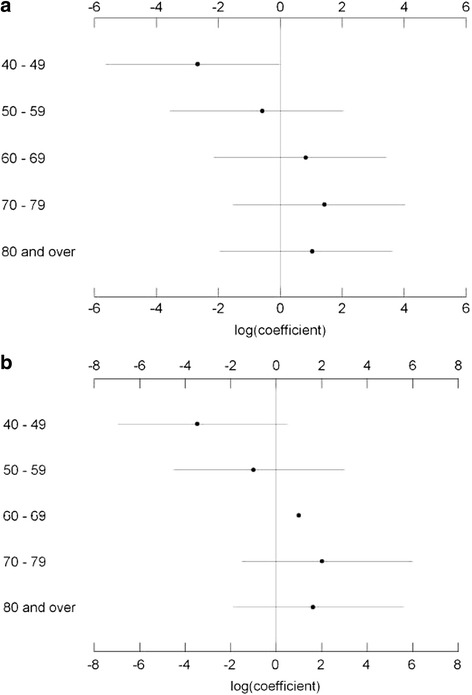
**a**: Unadjusted estimates and 95 % posterior intervals (95 % PI) for the Poisson regression (log) coefficients for the age group predictors (in 10-year age stratum) of primary total shoulder arthroplasty for males. **b**: Unadjusted estimates and 95 % posterior intervals (95 % PI) for the Poisson regression (log) coefficients for the age group predictors (in 10-year age stratum) of primary total shoulder arthroplasty for females

Figure 2a and b present the estimates and 95 % PI of the (log) coefficients for the age group predictors of TSA for males and females. Advancing age showed a proportional association with TSA for both sexes, however, these associations were variable with overlapping 95 % PI.

**Fig. 3:**
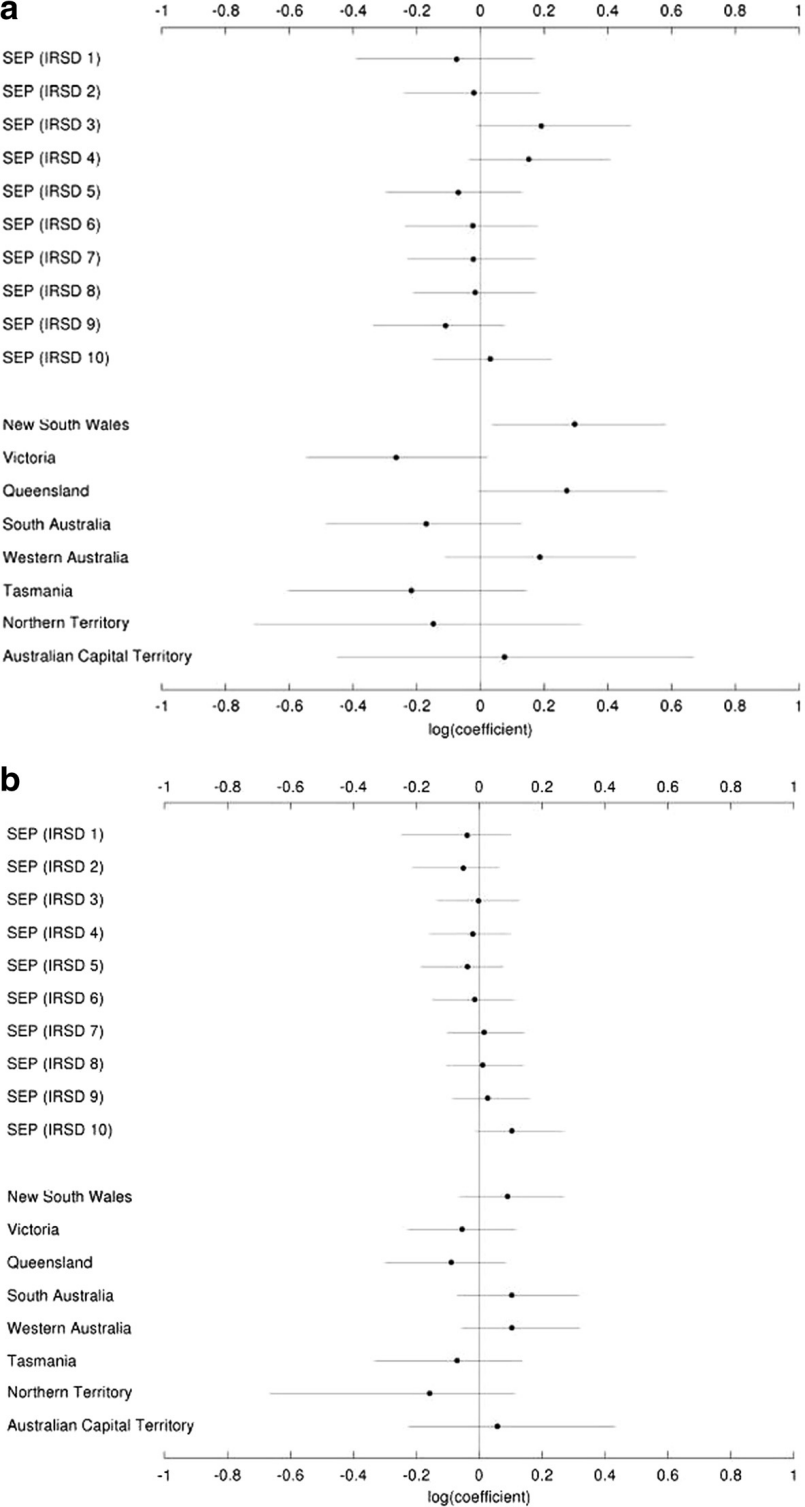
**a**: Estimates and 95 % credible intervals (95 % CI) for the Poisson regression coefficients for the local Government area (LGA)-level predictors, socioeconomic position (SEP) and State/Territory of primary total shoulder arthroplasty for males. **b**: Estimates and 95 % credible intervals (95 % CI) for the Poisson regression coefficients for the local Government area (LGA)-level predictors, socioeconomic position (SEP) and State/Territory of primary total shoulder arthroplasty for females

Figure 3a and b present the estimates and 95 % CI of the (log) coefficients for the LGA-level predictors of TSA, SEP deciles and State/Territory, for males and females; we present these (log coefficient) data graphed rather than tabulated to make for easier comparison of multiple estimates regarding the role played by SEP or geographic location on the uptake of TSA. These (log coefficients) estimates were also variable, with larger variations in SEP deciles observed for males as compared with females. Although large variations in TSA were observed at the State/Territory-level, these estimates were only statistically significant for men in New South Wales (0.29, 95 % CI 0.04–0.59). An opposite trend was observed between males and females for TSA uptake in South Australia. Furthermore, greater differences were observed at the State/Territory-level for males than for females, although, these were non-significant.

Table 1 presents the rate ratio (RR) estimates and 95 % CI in terms of the different in log (coefficients) relative to SEP decile 1. No differences in the ratio ratios of TSA were observed between any of the SEP deciles compared to decile 1 for either sex; compared to SEP decile 1 (most disadvantaged), the RR for SEP decile 10 was 0.11 for males (95 % CI–0.17, 0.45) and RR 0.14 for females (95 % CI–0.03, 0.42).

**Table 1:** Estimates (rate ratios) and 95 % credible intervals (95 % CI) for TSA relative to socioeconomic position (SEP) decile 1

SEP decile	Males	Females
1^a^	Referent	Referent
2	0.05 (−0.24, 0.39)	−0.01 (−0.21, 0.19)
3	0.27 (−0.04, 0.71)	0.04 (−0.14, 0.27)
4	0.23 (−0.06, 0.63)	0.02 (−0.16, 0.24)
5	0.01 (−0.30, 0.34)	0.00 (−0.20, 0.21)
6	0.05 (−0.25, 0.39)	0.02 (−0.16, 0.25)
7	0.05 (−0.24, 0.38)	0.05 (−0.12, 0.30)
8	0.06 (−0.23, 0.40)	0.05 (−0.12, 0.28)
9	−0.03 (−0.34, 0.27)	0.06 (−0.10, 0.32)
10	0.11 (−0.17, 0.45)	0.14 (−0.03, 0.42)

^a^ most disadvantaged SEP decile

Our posterior predictions sufficiently replicated the data generating process at the aggregated levels. Predicted rate ratios comparing SEP decile 10 (least disadvantaged) to SEP decile 1 (most disadvantaged) were centrally located in all predictions, with the positive predictive *p*-values being 0.4 for both models in males and 0.3 for both models in females. Positive predictions of aggregated TSA in each of the SEP deciles by State/Territory were appropriate for the larger States of New South Wales, Victoria and Queensland, with the other States/Territories being more variable in the posterior predictions.

## Discussion

Using the Australian National Joint replacement registry, with almost 100 % coverage, we found that associations between TSA uptake and SEP and region of residence appeared minimal. With regards to geographical regions of residence, the uptake of TSA was greater for men in New South Wales than any other State, and variations between regions were larger for males than females. Advancing age showed a proportional relationship with TSA, as would be expected.

Our observation is that few differences in TSA existed across SEP. Our findings of relative equity in TSA are similar to those of a US study that examined 3,529 TSA and which showed that insurance status (as a proxy for SEP) was not associated with uptake of TSA [40]. However, the lack of SEP variations in TSA are in contrast to the social gradient of arthritis in Australia, and also to the social gradient previously reported for the uptake of total knee arthroplasty across Australia [24]. Recent data indicates that individuals in the most socioeconomically disadvantaged areas of Australia are more likely to have doctor-diagnosed arthritis (at any skeletal site) compared to their less disadvantaged counterparts (7.3 % *vs.* 5.7 %, respectively) [16], thus our findings could indicate that TSA is being performed at a lower rate than expected. Given that our analyses excluded 7 % of patients in the AOA NJRR dataset due to unavailable SEIFA values (unavailable for reasons of low population density, amongst other reasons), it may be possible that those patients were from more disadvantaged areas, and thus our findings underestimate any potential SEP variation. For instance, it may be plausible that TSA is grossly underprovided to people residing in low density areas, however, the pain and immobility associated with OA is possibly better self-managed by those individuals with stoicism due to the difficulties associated with access and/or practitioner referral.

Our findings showed some geographic variation in TSA for both sexes although a greater variation was observed in males. Our data suggested that men from New South Wales were more likely to undergo a TSA than men from other States/Territories; there may be many possible reasons for that observation. One reason might be that New South Wales may have a greater predominance of employment in the mining or farming industries compared to other regions of Australia. Occupation-related activities associated with the mining and farming industries are explicitly more physically demanding than many other industries, and chronic overuse and trauma are correlated with an increased risk of developing shoulder OA [1, 41, 42]. However, our observations could be associated with the choice to utilise TSA prior to retirement. An alternative explanation for the greater uptake of TSA in New South Wales compared to other States/Territories is that there may be geographic variation in the number of surgeons, especially relative to the population in need.

We have previously shown that, compared to males, females are also more likely to undergo total knee arthroplasty [24, 43], although comparable uptake between the sexes was observed for total hip arthroplasty [44] as observed for TSA in this current study. Advancing age showed a proportional relationship with TSA; findings that are consistent with those from the US [13], and would be expected given the association between advancing age and OA prevalence [1, 16]. The peak of TSA uptake was observed in 70–79 year age group, which is indicative of OA being an age-related degenerative disease, although a lower TSA uptake was observed for those aged older than 80 years. Lower utilisation of TSA in older age groups may be explained by an avoidance of surgery so as to maintain the general health of the patient, or related to the patient being too ill to undergo anaesthesia and/or surgery, or where fewer functional gains may be expected post-surgery, due to higher levels of dependent care in these older populations. Despite the plausibility of those suggestions, positive outcomes post-surgery have been reported by a study of 50 TSA in 44 patients aged 80 years or older [45], and also in a study of 7 TSA performed in 6 patients aged 90 years of age or older with pre-operative forward elevation of >87 degrees [46]. Those data suggest positive outcomes and a greater ability to maintain independence with TSA, even at an older age in well selected populations.

Our study has various strengths. We present the first findings regarding associations between TSA and SEP and region of residence at the national level in Australia. Our analysis used data from a comprehensive register that is 99 % complete and is the largest shoulder arthroplasty registry in the world. We employed multilevel modelling which enabled us to investigate the complex and contextual effect of factors measured at various levels. Our study also has some limitations. Our data ascertainment from the AOA NJRR did not include hemi-arthroplasty because the use of this surgical intervention in Australia is more often employed for patients presenting with trauma-related injuries; thus these patients present with an alternative pathology to OA for whom TSA is more common. We did not investigate different types of surgical treatment, for instance arthroscopy, and are unable to comment on whether disease severity or waiting time differed prior to surgery across SEP or area of residence, as these data are not captured by the AOA NJRR. The greater variation seen in TSA uptake for men compared to women is likely an artefact of the smaller number of TSA in men. It was beyond the scope of these analyses to investigate whether any rural or regional *vs.* metropolitan differences exist in TSA, nor whether differences existed in relation to surgeons per capita. Finally, our indicators of SEP encompassed areas of ~250 households, and thus were not fine-grained; however, we do not believe this would be a potential confounder for our investigation regarding associations between area of residence and TSA uptake.

## Conclusions

In conclusion, our study used national registry data and showed that there were no variations in the uptake of TSA across SEP, nor were there geographic variations, with the exception of New South Wales. It is important that all countries monitor the equity of health service provision across SEP and geographic regions.

## Abbreviations

ABS, Australian Bureau of Statistics; AOA NJRR, Australian Orthopaedic Association National Joint Replacement Registry; IRSD, index of relative socioeconomic disadvantage; LGA, Local Government Area; OA, osteoarthritis; py, person years; SA1, statistical area level 1; SEIFA, SocioEconomic Index For Areas; SEP, socioeconomic position; TSA, total shoulder arthroplasty
